# Immunological Impact of Intestinal T Cells on Metabolic Diseases

**DOI:** 10.3389/fimmu.2021.639902

**Published:** 2021-02-18

**Authors:** Haiyan Zhou, Liwen Wang, Feng Liu

**Affiliations:** ^1^National Clinical Research Center for Metabolic Diseases, Metabolic Syndrome Research Center, and Department of Metabolism and Endocrinology, The Second Xiangya Hospital of Central South University, Changsha, China; ^2^Department of Pharmacology, University of Texas Health Science Center at San Antonio, San Antonio, TX, United States

**Keywords:** obesity, intestine, T cells, microbiota, dietary signals

## Abstract

Emerging evidence accumulated over the past several years has uncovered intestinal CD4^+^ T cells as an essential mediator in modulating intestinal immunity in health and diseases. It has also been increasingly recognized that dietary and microbiota-derived factors play key roles in shaping the intestinal CD4^+^ T-cell compartment. This review aims to discuss the current understanding on how the intestinal T cell immune responses are disturbed by obesity and metabolic stress. In addition, we review how these changes influence systemic metabolic homeostasis and the T-cell-mediated crosstalk between gut and liver or brain in the progression of obesity and its related diseases. Lastly, we highlight the potential roles of some drugs that target intestinal T cells as a therapeutic treatment for metabolic diseases. A better understanding of the interaction among metabolites, bacterial signals, and T cell immune responses in the gut and their roles in systemic inflammation in metabolic tissues should shed new light on the development of effective treatment of obesity and related disorders.

## Introduction

Obesity is a key risk factor for many chronic diseases and represents a growing global and serious public health crisis. Recent studies show that individuals with obesity are linked with a great increase in morbidity and mortality from COVID-19 infection (1, 2). Obesity is characterized by low-grade chronic inflammation in key metabolic tissues such as adipose tissues, which control whole-body energy homeostasis (3). The intestinal tract, continuously exposed to dietary factors and foreign antigens, is a critical site of high immune challenge. A complex and highly specialized network of innate and adaptive immune cells is orchestrated to deal with this complex situation, including the largest population of T cells in the body (4). In recent years, emerging evidence has revealed an integral link between the microbial or dietary signals and gut T cell immune responses during obesity development (5–8).

Conventional gut-resident T cells mainly include CD8^+^ T cells and CD4^+^ T cells, the latter are generally composed of T-helper 1 (Th1) cells, T-helper 2 (Th2) cells, T-helper 17 (Th17) cells, Follicular helper T (Tfh**)** cells, and regulatory T (Treg) subsets (4, 9). Th17 cells and Treg cells are the most abundant CD4^+^ T cells in mucosal tissue. Th17 cells, induced by TGF-β and IL-6 through the master transcription factor RORγt, play a critical role in host defense against fungi and maintenance of intestinal homeostasis through producing IL-17 and/or IL-22 (10, 11). However, aberrant activation of Th17 cells could lead to the pathogenesis of various autoimmune diseases (12). CD25^+^Foxp3^+^ Treg cells play a nonredundant role in the maintenance of intestinal homeostasis in a IL-10 and TGF-β-dependent mechanisms (11). Th1 cells, which are a major source of IFN-γ, are important mediators in the eradication of intracellular pathogens such as viruses and bacteria (13). Tfh cells, characterized by their expression of B cell follicular homing chemokine receptor CXCR5 and co-stimulatory molecules PD-1 (14, 15), help differentiation of germinal center B-cells and production of high-affinity antibody including intestinal IgA (16). In addition to the widely studied conventional CD4^+^ and CD8^+^ T cell subsets, the gut is enriched by unconventional T cells, including the γδ T cells, natural killer T (NKT) cells, and mucosal-associated invariant T (MAIT) cells. All these cells are critical regulators in maintaining gut barrier function and immune homeostasis (17, 18). The potential role of these unconventional T cells in intestinal immunity and inflammation has been reviewed elsewhere (19–21). In the current review, we focus on the crosstalk between dietary- and microbiota-derived signals and intestinal T cell immune responses in the regulation of obesity and its related disorders. We also highlight the roles of intestinal T cells in mediating the communication between the gut and other organs in the initiation and progression of metabolic-related diseases. Ultimately, we underline an emerging concept that modulating gut T cells may be an effective approach in treating obesity-induced metabolic diseases.

## Intestinal T-Cell Anatomical Distribution

The small and large intestines, which digest and absorb nutrients and water from ingested food, comprise a continuous tube that stretches from the outlet of the stomach to the anus. In the small intestine, the finger-like projections called villi drastically increase the surface area of the small intestine for greater absorption of the digested food. In the colon, villi are absent and the epithelium surface is flat with smaller crypts, which correlates with their function as reabsorbing water from feces and acting as a barrier to the commensal microbiota. Gut homeostasis is maintained by the intestinal epithelial barrier, mucus layer, commensal microbiota as well as the gut immune system (22).

**Figure 1:**
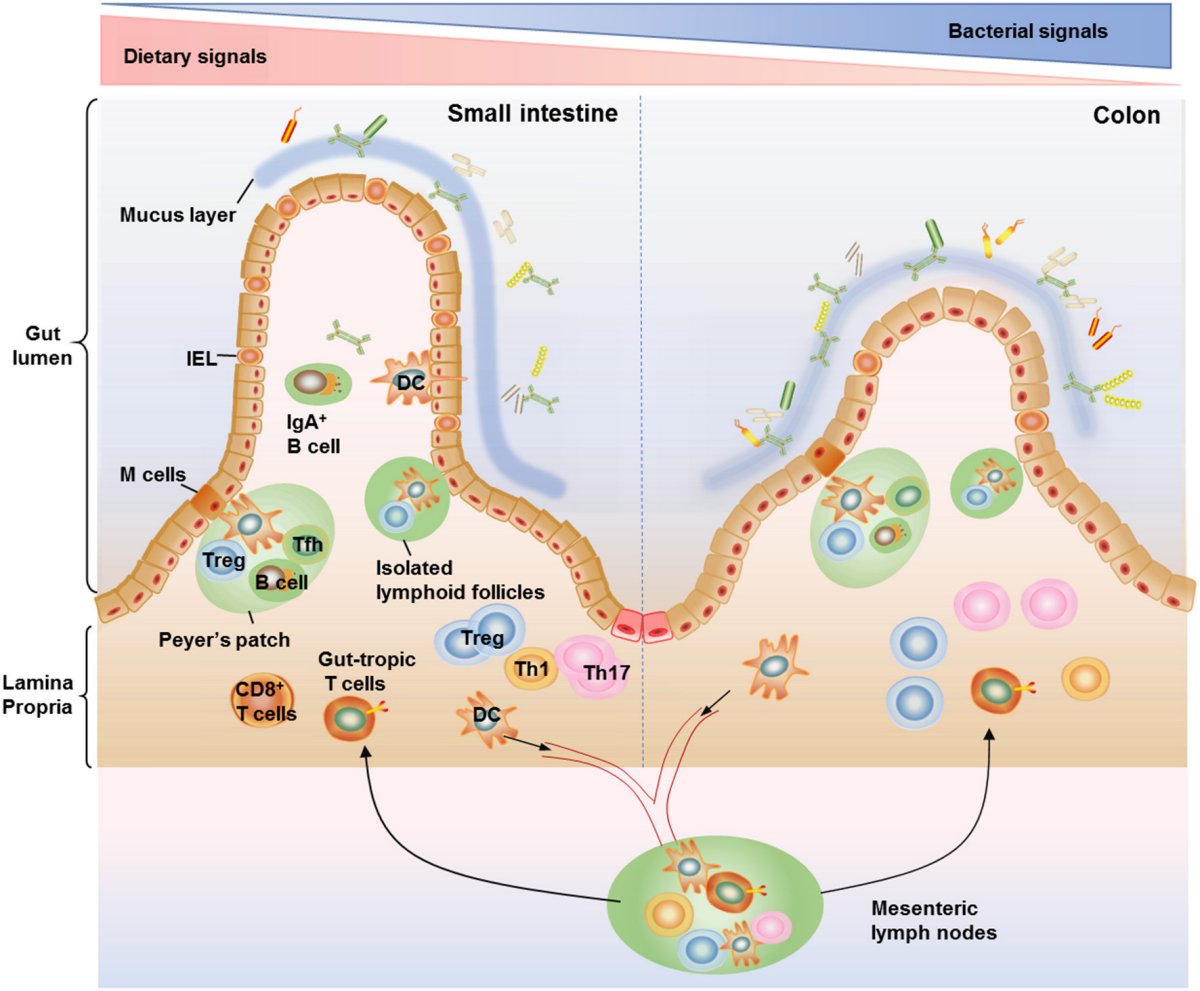
Anatomical distribution of intestinal T cells. T cells distributed within the small and large intestine are frequently arranged within the gut-associated lymphoid tissue (GALT), which are composed of organized lymphoid tissues including mesenteric lymph nodes, Peyer's patches, and isolated lymphoid follicles (ILFs) as well as more diffusely scattered lymphocytes including the lamina propria lymphocytes and intraepithelial lymphocytes (IELs). IELs reside within the epithelium layer and are proximity to antigens in the gut lumen, making them components of the front line of immune defense against invading pathogens. There are more IELs in the small intestine compared with the colon. The majority of T cells in lamina propria are CD4^+^ T cells, with only a small population of CD8^+^ T cells. Among the CD4^+^ T cell subsets, Th17 cells and Treg cells are the most abundant cells in the gut, conferring protection against fungi and maintenance of intestinal homeostasis. The organization of Peyer's patches is comparable to that of lymph nodes, with large B cell follicles and T cell areas. Peyer's patches are in close contact with microfold cells (M cells) located in gut epithelium which can capture and transport antigens from the lumen to antigen-presenting cells such as dendritic cells (DCs) in the underlying Peyer's patches. DCs can also form transepithelial dendrites to directly sample luminal antigens and then emigrate to the mesenteric lymph nodes, where they activate naive T cells to induce gut-tropic T cells. The ILFs, with features similar to Peyer's patches, are distributed along the whole intestinal tract and believed to provide a complementary system for Peyer's patches.

T cells distributed within the small and large intestine are frequently arranged within the gut-associated lymphoid tissue (GALT), which are composed of organized lymphoid tissues and more diffusely scattered lymphocytes (23). The organized lymphoid tissues include mesenteric lymph nodes, Peyer's patches, isolated lymphoid follicles (ILFs), and the diffusely scattered lymphocytes consists of intraepithelial lymphocytes (IELs) compartment and the lamina propria lymphocytes (LPL) compartment (24, 25). Peyer's patches occur in the fetal small intestine independent of the intestinal flora (25). The organization of Peyer's patches is comparable to that of lymph nodes, with large B cell follicles and T cell areas. Peyer's patches are in close contact with microfold cells (M cells) located in gut epithelium which can capture and transport antigens from the lumen to antigen-presenting cells such as dendritic cells (DCs) in the underlying Peyer's patches (26). DCs can also form transepithelial dendrites that enable the cells to directly sample luminal antigens (27, 28). These antigen-loaded DCs emigrate through lymphatics to the mesenteric lymph nodes, where they present the captured antigens to T cells (28). The ILFs, with features similar to Peyer's patches (29), are distributed along the whole intestinal tract, and, unlike Peyer's patches, their development is triggered by the intestinal flora (30). IELs are in direct contact with the enterocytes and proximity to antigens in the gut lumen, which making them components of the front line of immune defense against invading pathogens (31). There are more IELs in the small intestine compared with the colon (32). IELs are usually CD8^+^ T cell populations with a significant proportion of γδ TCR (33, 34). By contrast, the majority of the T cells in lamina propria are CD4^+^ T cells, with only a small population of CD8^+^ T cells in this location (35). Similar to their distribution within the IELs, lamina propria CD4^+^ T cells are accumulated at higher levels within the colonic than within the small intestine (11). The vast majority of T cells present in the gut epithelium and lamina propria are antigen-experienced effector/memory phenotypes, making them suitable to quickly deal with both harmless and hazardous stimuli from incoming antigens (35) (Figure 1).

## The Intestinal T Cell Immune Responses Influenced by Obesity-Altered Microbial and Dietary Signals

The adaptive responses of T cells in the intestine, which are influenced by microbial and dietary factors associated with diet-induced obesity, are critical regulators in systemic inflammation and glucose metabolism.

### Overview of Intestinal T Cells in the Context of Obesity

High fat diet (HFD) feeding induces changes in intestinal immunity before the onset of systemic low-grade inflammation and insulin resistance (5). It has also been shown that the effects of HFD on intestinal T cells are more confined in the small intestinal lamina propria (SILP) while little or no change is observed in the colon, suggesting that the interaction between the immune system and gut microbiota localized in the SILP is crucial in the development of metabolic disease (5, 36).

Contrary to unaltered Th2 cells in the gut of obese mice, HFD feeding leads to an increase in the proportion of IFN-γ^+^ Th1 cells and CD8^+^ T cells in the SILP (5–7). Likewise, T-bet^+^ Th1 cells are increased in the small intestine and colon of obese patients compared with lean control human subjects (6). IFN-γ^−/−^ mice display improved barrier function compared to wild-type (WT) mice under HFD feeding conditions, implicating that local intestinal IFN-γ production may be one of the critical mediators of intestinal permeability in obesity (6). Consistent with this finding, reduced infiltration of IFN-γ-producing cells in the bowel contributes to the improved gut barrier function of β7-deficient mice under HFD feeding conditions (37).

HFD feeding reduces the proportions and numbers of RORγt^+^Th17 and Treg cells in the SILP (5–7). Consistently, Treg cells are decreased in the small intestine and colon of obese patients compared with lean control human subjects (6). It is suggested that the reduction of *Porphyromonadaceae*, a family within the order Bacteroidales, in the ileum at the onset of HFD feeding could be responsible for the reduction of Th17 cells (5). Co-transfer of Th17 and Treg cells lowers the fasting glucose and insulin levels, improves glucose tolerance and insulin sensitivity in obese Rag1^−/−^ mice, while transfer of Treg cells alone has no effect (7). Consistently, Rag1^−/−^ mice transferred with T cells from RORγt^−/−^ mice show increased body weight gain, insulin resistance, and hyperinsulinemia compared to those injected with T cells from WT mice even under normal chow diet feeding conditions (5). Induction of IL-22, a major effector cytokine of Th17 cells (38), is impaired in the colon of obese mice during infection (39). In line with this finding, mice deficient in IL-22 receptor are prone to developing metabolic disorders under HFD feeding conditions (1). Accordingly, administration of exogenous IL-22 into genetically obese mice and HFD-fed mice reverses many of the metabolic dysfunctions (1, 40). IL-17 is another critical cytokine produced by Th17. Increasing IL17-expressing cells in the intestine by pretreating mice with dextran sodium sulfate, which increases gut permeability to bacteria and elevates IL17 production, prevents HFD-induced glucose intolerance and insulinopenia (5). In accordance with a decrease in intestinal Th17 cells in mice, T2D patients are more sensitive to intestinal infections with pathogens like *Candida guilliermondii* compared to healthy controls (41, 42). Notably, the decrease of Th17 cells and IL-17 levels appears to be intestine-specific as elevated IL-17 levels are observed in peripheral blood, adipose tissues, and livers of obese subjects (43–45), suggesting that Th17 cells may have distinct function in the intestine from that in other tissues. Consistent with this, obesity-induced expansion of Th17 cells in the spleen is associated with inflammatory autoimmune diseases in the brain (46, 47). Taken together, these findings suggest that obesity differentially regulated the immune responses of T cell subsets in the intestine, leading to disturbed gut homeostasis.

### Communications Between Gut Microbiota and T Cell Immune Responses

The number and type of bacteria in the gastrointestinal tract vary by region, ranging from about 10^5^ per ml in the upper small intestine and up to 10^12^ per ml in the colon (48). It is well established that obesity and T2D are associated with an increase in the ratio of *Firmicutes* to *Bacteroidetes* phyla in the gut microbiota (49, 50). The causality of gut microbiota in the development of metabolic diseases is demonstrated in rodents by the finding that microbiota transplantation from obese mice or humans to germ-free recipient mice is sufficient to induce obesity phenotypes (50–52). In addition to its regulation in lipid metabolism (53–55), gut microbiota may regulate obesity by influencing the innate and adaptive immune response (56).

Intestinal microbiota plays key roles in mediating T cell function in the gut (57). Th17 cells are absent in the intestine of both germ-free mice and specific pathogen-free animals obtained from Jackson Labs (58). Segmented filamentous bacteria (SFB), which induces serum amyloid A (SAA) that stimulates lamina propria DCs and promotes the development of Th17 cells in the gut (59–61), is much less abundant after 10 and 30 days of HFD feeding (5). Ileum microbiota transplantation experiments further indicate that HFD-induced changes in the gut microbiota may be the direct cause of the decreased Th17 cells in the ileum, which is correlated with increased metabolic dysfunction (5). On the other hand, re-establishment of gut-tropic Th17 cells in the small intestine of obese Rag1-deficient mice results in a significant increase of *Bacteroidetes* and a decrease of *Firmicutes*, both changes are associated with leanness (7). Furthermore, a mucin-degrading bacterium *Akkermansia muciniphila*, which is found to prevent obesity-induced metabolic disorders (62, 63), is also increased after Th17 cell transfer (7). Gut-tropic Treg cells, which express high levels of CCR9, CD103, and killer cell lectin-like receptor G1 (KLRG1), are mostly abundant in the intestine mucosa (64). A significant decrease in the number of Foxp3^+^ Tregs was observed in the colonic but not SILP, of germ-free mice or antibiotic-treated mice (65, 66). These results are consistent with previous observations that germ-free mice have increased proportions of Tregs in small intestine (58), suggesting a differential requirement of microbiota for the induction and maintenance of Treg cells between the small intestine and colon. It is suggested that activation of CD4^+^ T cells in the small intestine is driven mainly by dietary antigens whereas in the colon it is induced by the microbiota (67). Indeed, colonic Treg cells possess unique T-cell receptors (TCRs) different from those used by Treg cells in other organs, further implying an important role for local microbial antigens in shaping the colonic Treg cell population (68). Nevertheless, colonization (inoculated by gavage) of mice with a defined mix of *Clostridium* strains provides a TGF-β-rich environment that upregulates the IL-10-producing colonic Treg Cells (66). Colonization of germ-free mice with *Bacteroides fragilis* (*B. fragilis*) promotes the differentiation and function Foxp3^+^ Treg via its immunomodulatory molecule polysaccharide A (PSA), where Toll-like receptor 2 (TLR2) signaling is required for both Treg induction and IL-10 expression (69).

Tfh cells, which are localized in the germinal centers of Peyer's patches and mesenteric lymph nodes, promote class switch and somatic hypermutation in germinal center B cells to produce high-affinity IgA (70). Germ-free mice or mice treated with an antibiotic cocktail show impaired Tfh development within the gut and this microbiota-dependent Tfh development relies on the T-cell intrinsic MyD88 signaling (16). T cell-specific knockout of myeloid differentiation primary response 88 (MyD88) results in abnormal IgA antibody responses and an altered microbial gut community, leading to more severe inflammatory diseases, age-associated obesity, and metabolic disorders (16, 53). On the other hand, oral treatment of germ-free mice with a purified TLR2 agonist alone was capable of significantly increasing germinal center-Tfh abundance within Peyer's patches (16). In summary, these findings raise the possibility that modulating the interplay between microbiota and intestinal T cell immune responses may provide new treating strategies in metabolic diseases.

### The Dietary Signals That Influence Intestinal T Cell Immunity

Nutritional status potentially influences immune responses. How dietary signals regulate immune cell dynamics and function has gradually aroused great interest in recent years.

#### Bile Acids

Bile acids are cholesterol-derived natural surfactants abundant in the mammalian gut, where they undergo bacteria-mediated transformation to generate a large pool of bioactive molecules that are critical for lipid digestion, antibacterial defense, and glucose metabolism (71, 72). Pharmacologic stimulation of bile acid receptors, which are mainly expressed on immune cells, prevents obesity by decreasing blood glucose levels and increasing insulin sensitivity (73). By contrast, compared with germ-free mice colonized with WT *Bacteroides thetaiotaomicron*, germ-free mice colonized with the bile salt hydrolase-depleted *Bacteroides thetaiotaomicron* display reduced body weight gain on a HFD, decreased fat accumulation in blood and liver, and enhanced energy expenditure due to a selective alteration of bile acid pools (74). Lithocholic acid, a secondary bile acid metabolite, impedes Th1 activation by decreasing the production of Th1 cytokines IFN-γ and TNF-α (75). Recently, two distinct derivatives of lithocholic acid, 3-oxoLCA and isoalloLCA, have been found to impair the differentiation of Th17 cells and increase the differentiation of Treg cells, respectively (76). Combined treatment of mice with these two bile acid metabolites skews T cells into Treg cell at the expense of Th17 cells in the intestinal lamina propria (76). Since bile acids exist in the gut where abundant Treg and Th17 reside, it would be of great interest to determine the functional roles of these bile acid derivatives in metabolic diseases.

#### Retinoic Acid

Retinoic acid, a major metabolite of Vitamin A, is found at higher concentrations in the small intestine and the mesenteric lymph nodes compared with the colon (77). Retinoic acid regulates the intestinal immune homeostasis via generating gut-homing effector T cells and induction of Treg cells (77–79). Retinoic acid is also required to elicit pro-inflammatory CD4^+^ T cell responses to infection and mucosal vaccination, since blocking retinoic acid receptor signaling results in a cell-autonomous impairment in CD4^+^ T cell activation (61). Additionally, depletion of vitamin A in obese mice further reduced the proportion of Th17 cells in the small intestine, leading to increased body-weight gain and insulin resistance, while adoptive transfer of *in vitro*-differentiated gut-tropic Th17 cells to obese mice ameliorates these metabolic disorders (7). These findings reveal a critical role of retinoic acid in T cell function in the regulation of metabolic consequences.

#### Short-Chain Fatty Acids

Short-chain fatty acids, including acetic, propionic, and butyric acid, are generated in larger quantities through fermentation of sugars, proteins, and soluble fibers (80). Signals from short-chain fatty acids could stimulate metabolite-sensing G protein-coupled receptors (GPR), which are generally expressed on gut immune cells and some gut epithelial cells (81). Among them, GPR43 on Treg cells and GPR109 on DCs appear to be critically important for gut homeostasis. Treg differentiation and its suppressive function are abolished in GPR43-deficient and GPR109-deficient mice (82, 83). Mice fed a HFD with supplement of butyric acid display a significant increase in intestinal Treg generation and energy expenditure (84, 85). However, higher concentrations of butyrate has been found to induce the expression of Th1 master transcription factor T-bet (86), suggesting that butyrate may exert either beneficial or detrimental effects on the mucosal immune system depending on its concentration and immunological milieu.

#### Dietary Salt

Excessive intake of dietary salt, which is highly contained in the western diet, can lead to hypertension, one of the major complications of obesity (61). Mice fed with a high-salt diet (HSD) for 3 weeks exhibit higher frequencies of lamina propria Th17 cells compared to normal chow-fed mice via inducing salt-sensing kinase serum glucocorticoid kinase-1 (SGK1) (40). However, these promoting effects are abolished in germ-free mice, indicating a crucial role for intestinal bacteria in mediating the effect of a HSD on Th17 cells (60). Reduced amount of *Lactobacillus murinus* (*L. murinus*) may contribute to the increased frequencies of lamina propria Th17 cells within the small intestine and colon (60). In line with this, colonization of germ-free mice with *L. murinus* could significantly reduce the frequencies of lamina propria Th17 cells induced by SFB (60). HSD also disturbs intestinal homeostasis by attenuating Treg function, either promoting IFN-γ secretion from human Treg cells or decrease luminal levels of Treg-inducing butyrate (43, 50).

#### Aryl Hydrocarbon Receptor (AhR)

AhR, a widely expressed basic helix-loop-helix transcription factor that is abundantly expressed on murine IELs (87), can be activated by ligands from fruits, nuts, and cruciferous vegetables. AhR activation promotes gene expression of mediators involved in the regulation of gut homeostasis; such mediators include IL-22, anti-microbicidal factors, and increased Th17 cell polarization (81). Alternatively, it is also suggested that AHR regulates both Treg and Th17 cell differentiation in a ligand-specific fashion (88). Lack of AhR signaling in IELs compromises the maintenance of IELs and the control of the microbial load and composition, leading to reduced immune surveillance and increased vulnerability to epithelial damage (87, 89). However, a quantitative trait locus analysis of dietary obesity in C57BL/6 and 129P3/J F2 mice revealed that the AhR gene is one of the seven candidate genes associated with increased body weight (90). Consistently, AhR^−/−^ mice are protected against diet-induced obesity and glucose intolerance (91, 92). These results suggest that T cell-specific, but not systemic, ligation of AhR may provide beneficial effects on defending obesity.

Taken together, although this list of dietary signals on gut T cell performance is not exhaustive, one can postulate that modulating dietary factors may greatly influence intestinal T cell function and consequently gut homeostasis.

## Intestinal T Cells Under Other Metabolic Stresses

### Aging

The number of IELs both in the small and large intestines are highest in 6-month-old mice and then gradually decreases with age, which may be one of the aging phenomena of the intestinal immune system that increase liability to intestinal infections (93). Th2 immune responses against gastrointestinal nematode parasites are compromised in aging mice, due to inappropriate or insufficient activation of CD4^+^ T cells in the submucosa (94). T cell-specific ablation of MyD88 impairs Tfh cell development and IgA production within the gut, leading to age-associated obesity (53). It is suggested that altered gut microbiota and increased lipid absorption are responsible for T cell-mediated regulation of age-associated metabolic disorders (53). Moreover, compared with younger people (<45 years), the small intestinal CD4^+^ T cells from older human subjects (>65 years) display altered phenotypic and functional profiles including reduced expression of a co-inhibitory molecule, increased spontaneous cell death, and both reduced frequencies and altered functional responses of specific T cell subsets (95). These changes may contribute to altered intestinal homeostasis and loss of protective gut immunity with age.

### Food Availability

It has been postulated that early childhood malnutrition confers life-long immunodeficiency with an increased risk for metabolic diseases such as cardiovascular disease and insulin resistance (96, 97). Although the underlying mechanisms remain unclear, it is proposed that defects in the diversity and composition of commensal microbes as well as impaired gut immune function in response to malnutrition may contribute to these outcomes (96, 98–100). Consistently, mice weaned onto macromolecule-depleted chow lack peripherally generated Treg cells in the small intestinal required for oral tolerance (67). On the other hand, many studies have defined the beneficial effects of caloric restriction on metabolic diseases (101, 102). Intermittent fasting (IF) decreases Th17 but increases Treg cells in the SILP, which, along with enriched beneficial gut bacteria, contribute to the ameliorated experimental autoimmune encephalomyelitis (EAE), a mouse model of multiple sclerosis diseases (103). However, compared with those of *ad libitum*-fed juvenile mice, longer time fasting (>36 h) greatly reduced the numbers of IgA^+^ B cells as well as CD4^+^ and CD8^+^T cells in Peyer's patches, leading to the failure in inducing oral tolerance (104). Consistently, a time-restricted feeding regimen in juvenile mice exacerbated metabolic disorders (105). These findings suggest that factors such as age may affect the outcome of caloric restriction. Nevertheless, it remains to be determined as to how gut T cell immune systems are affected by caloric restriction or malnutrition and to what extent these changes in gut T cells contribute to the physiological outcome in adult mice.

## Crosstalk Between Antigen-Presenting Cells (APCs) and T Cells in the Intestine

Small intestinal APCs are believed to sample and present commensal bacteria to the gut-associated T cells to maintain immune homeostasis (106). Two major populations of intestinal APCs have been identified based on differential expression of the integrin subunit CD103 and the chemokine receptor CX3CR1 (107). Under steady state, intestinal tolerogenic CD103^+^ DCs, which are dispersed throughout the lamina propria and can migrate to the draining the mesenteric lymph nodes, are potent generators of Treg cells through their ability to activate TGF-β and metabolize vitamin A into RA, the latter also underlies the enhanced capacity of CD103^+^ DCs to induce the gut-homing T cells (77, 108, 109). DCs that lack integrin αvβ8, one of the major activators of Treg-inducing TGF-β, led to the loss of Foxp3^+^ Tregs in the lamina propria (110). CD103^+^ DCs can also acquire inflammatory properties during intestinal inflammation such as the ability to produce IL-6 and drive Th1 and Th17 responses (111, 112). CX3CR1^+^CD103^−^APCs, which are composed of DCs and macrophages and populate the lamina propria of the intestine, can form transepithelial dendrites that enable the cells to directly sample luminal antigens (27). A subset of these CX3CR1^+^CD103^−^APCs, identified as CD70^high^CD11c^low^ cells, expresses Th17-inducing molecules in response to commensal organism-derived ATP stimulation and preferentially induces Th17 differentiation (113). These observations highlight the importance of commensal bacteria and ATP for Th17 differentiation in health and disease and offer an explanation of why Th17 cells are merely found in the mesenteric lymph nodes and Peyer's patches (113, 114). Though HFD did not induce obvious changes in the proportions of CD103^+^ DCs or CX3CR1^+^ mononuclear phagocytes, it enhanced the ability of CD103^+^ DCs to induce Th1 differentiation while inhibiting the ability of CX3CR1^+^ cells to induce Th17 differentiation (7). Genes involved in T cell co-stimulation such as *Icam1* and *Cd86* and Th17-inducing cytokines such as IL-6 and IL-12p40 are all downregulated in the SILP APCs after 30 days HFD feeding, which is consistent with an early significant decrease of Th17 cells in the small intestine (5). In summary, these HFD-induced changes in APC characteristics correlate well with the increased Th1 cells and reduced Th17 cells observed in small intestine of HFD mice, adding a potential way to modulate T cell immune responses via manipulating APCs.

## Intestinal T Cell-Mediated Crosstalk Between Gut and Other Tissues

Obesity is associated with an impaired intestinal barrier (49), which causes increased translocation of food antigen, bacterial components, and bacterial metabolites from the gut to various metabolic tissues such as liver and brain, triggering local immune responses (49, 115). Obesity is reported to be associated with higher Parkinson's disease risk among never smokers (116). It has been proposed that initial α-synuclein aggregation and subsequent Lewy bodies generation in the gut occurred several months before the manifestation of motor symptoms (117). Dysbiosis could lead to an oxidative environment, where oxidized α-synuclein are captured by mucosal DCs and activate intestinal pro-inflammatory Th1 and Th17 cells that play a fundamental role in promoting nigrostriatal neurodegeneration (117, 118). In addition to Parkinson's disease, growing evidence suggests that obesity is associated with the susceptibility and disease severity of multiple sclerosis (119). In an adoptive-transfer EAE model, the transferred Th17 cells preferentially infiltrate into colonic lamina propria and enter blood circulation via lymphatic vessel. Preventing Th17 cells from entering into the colon significantly attenuates EAE (120). Non-alcoholic fatty liver (NAFLD) is strongly associated with immune turbulence in the mesenteric lymph nodes, with increased ratios of Th1/Th2 cells and Th17/Treg cells (121). These gut-derived memory T cells could migrate to the liver and promote liver inflammation (122–124). Restoration of ratios of the mesenteric lymph node CD4^+^ T cell subsets markedly alleviates NAFLD progression (121). These studies suggest that intestinal T cell immune responses may play a critical role in mediating inflammatory brain or liver diseases, whereas the detailed mechanisms are remained to be elucidated.

## Drugs Targeting Intestinal T Cells in Treating Obesity and Its Related Complications

HFD feeding induces an early pro-inflammatory shift in gut immune responses, which contributes to a later-stage systemic low-grade inflammation and obesity-related insulin resistance. Thus, developing treatments that are restricted to the gut inflammation could avoid broad systemic deleterious effects and be an effective approach for metabolic disease (6).

5-aminosalicylic acid (5-ASA), first-line therapy for inflammatory bowel disease (IBD) in humans (125), contributes to improved systemic metabolic parameters in mice during HFD feeding through changes in intestinal barrier function, fat inflammation, and oral tolerance to gut luminal antigen (6). Specifically, 5-ASA reduces IFN-γ-producing Th1 and CD8^+^ T cells while promotes Treg cell accumulation in the intestine, leading to alleviated gut and even visceral adipose tissue inflammation in mice (6). Pituitary adenylate cyclase-activating polypeptide (PACAP), a neuropeptide well-known for its functions in inhibiting inflammation, decreases the number of apoptotic epithelial T cells in the ileal and colon of mice (126). In addition, a novel probiotic mixture, Prohep, inhibits liver tumor growth in mice by downregulating Th17 cells in the small intestine which could otherwise contribute to liver tumor growth after migrating to the liver via the cardiovascular system (127). NX-13, an orally active, gut-restricted novel drug (128), decreases the differentiation of Th1 and Th17 subsets in an NLRX1-dependent manner *in vitro*, which contributes to alleviated disease severity of mouse IBD models (129). Besides, BT-11, an investigational new drug for IBD, has been found to alleviate IBD by inducing Treg cells in the mouse colonic lamina propria through controlling glucose flux and enhancing IL2/STAT5 signaling axis (130, 131). It has been shown that oral anti-CD3 mAb is absorbed in gut-associated lymphoid tissue and stimulates CD4^+^CD25^−^LAP^+^ Tregs to suppress autoimmune diseases (132), like autoimmune diabetes in NOD mice (133). Oral anti-CD3 plus β-glucosylceramide (an NKT cell target antigen) treatment promotes Treg cell generation in mesenteric lymph nodes and bowel and alleviates inflammation in adipose tissue and improves hepatic steatosis in ob/ob mice (134). Accordingly, non-alcoholic steatohepatitis patients with oral OKT3 (anti-CD3 antibody) shows increased Treg cell proportions and reduced AST and fasting plasma glucose levels and alleviated insulin resistance (32). Taken together, these studies suggest that targeting gut T cells holds huge potential in treating obesity and its associated diseases (Table 1).

**Table 1:** Drugs targeting intestinal T cells in treating obesity and its related complications.

**Drug**	**Disease model**	**Dosage**	**Intestinal T cell**	**Metabolism**	**References**
5-ASA	Diet-induced obesity (*n* = 10–15)	1,500 or 150 mg/kg/day for 12–14 weeks	Reduce IFN-γ-producing T cells Increase Treg cells	Alleviate Gut and VAT inflammation, improve glucose tolerance, and insulin sensitivity	(6)
PACAP	Subacute ileitis model (*Toxoplasma gondii* infection, *n* = 21–26)	1.5 mg/kg/day for 6 days from day 3 post-infection (p.i.) until day 8 p.i	Decrease T cells in ileal and colon	Alleviate intestinal inflammation	(126)
Probiotics (Prohep)	s.c. HCC model (*n* = 6–8)	Starting 1 week before or at the same day until 38 days after tumor injection	Downregulate Th17 cells	Inhibit the liver tumor growth	(127)
NX-13	Adoptive transfer colitis (*n* = 10) Spontaneous colitis by Mdr1a^−/−^ (*n* = 9) Dextran sodium sulfate colitis (*n* = 9)	0, 1, 10, 20 mg/kg/day for 6–8 weeks DSS group for 7 days	Decrease Th1, Th17 subsets	Suppress intestinal inflammation	(129)
BT-11	Adoptive transfer colitis (*n* = 10) DSS colitis (*n* = 10)	8 mg/kg/day for 6 weeks DSS group for 7 days	Induce Treg cells	Alleviate intestinal inflammation	(130)
anti-CD3 mAb	Genetic obesity (*n* = 10)	5 μg of anti-CD3 Plus 100 μg of GC for 5 days	Promote Treg generation	Alleviate inflammation in adipose tissues Reduce hepatic steatosis	(134)
	Biopsy-proven NASH patients (*n* = 9)	0.2, 1.0, 5.0 mg/day for 30 days		Reduce AST and fasting plasma glucose level	(32)

## Conclusions and Perspectives

**Figure 2:**
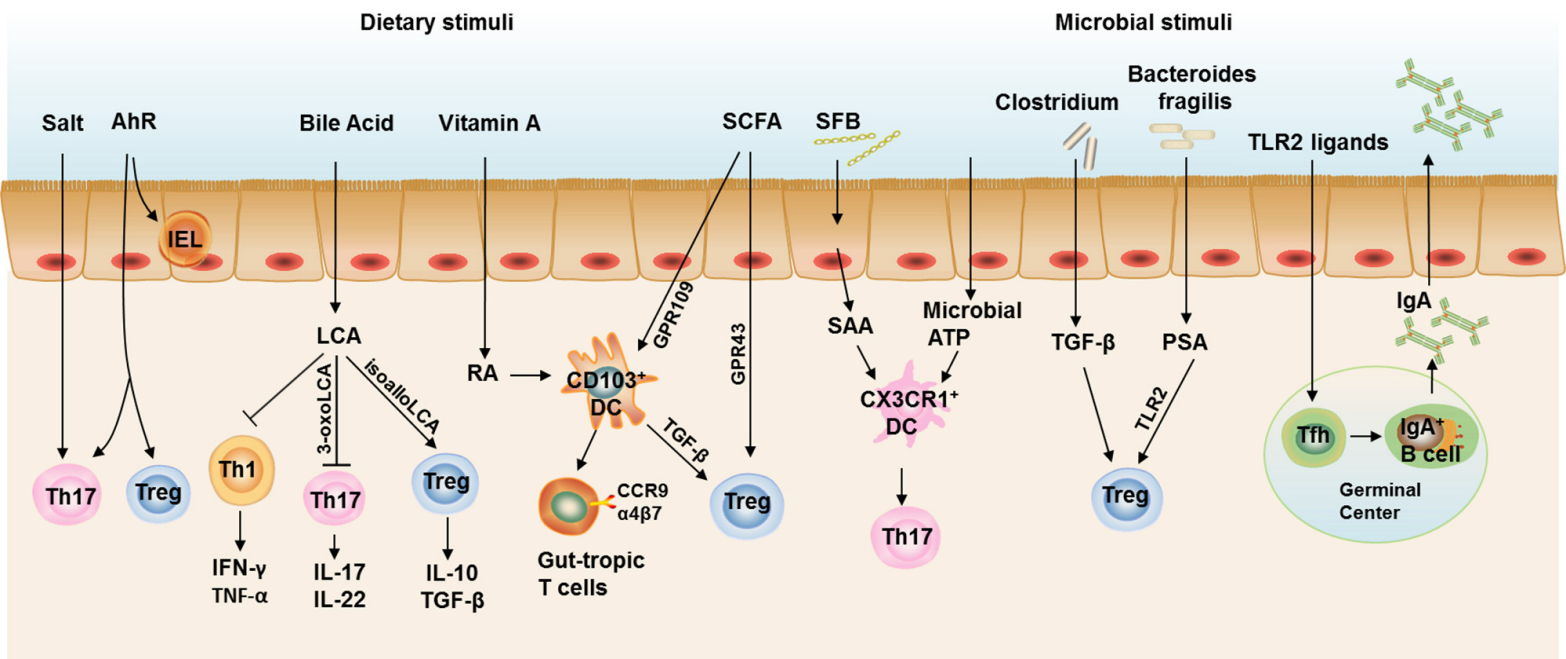
Intestinal T cell immune responses under steady state. Th17 cells and Treg cells are the most abundant CD4^+^ T cells in the gut to maintain intestinal homeostasis. Excessive salt intake and AhR ligation both contribute to the increased Th17 cell polarization in the gut, while AhR ligation also required for the induction of Treg cells and maintenance of IELs. Bile acids are cholesterol-derived natural surfactants abundant in the mammalian gut. Lithocholic acid, a secondary metabolite of bile acids, impedes Th1 activation and two distinct derivatives of lithocholic acid, 3-oxo-lithocholic acid and isoallo-lithocholic acid, have been found to impair the differentiation of Th17 cells and increase the differentiation of Treg cells, respectively. CD103^+^ DCs represent the dominant DC population in the murine small intestinal lamina propria (SILP). CD103^+^ DCs induce the gut-homing receptors CC chemokine receptor (CCR)9 and α4β7 on responding T cells and Treg cell differentiation, both of which are dependent on signaling events initiated by the vitamin A metabolite, retinoic acid (RA). Short-chain fatty acids (SCFAs) could also induce Treg polarization directly through a receptor GPR43 on T cells or indirectly through a receptor GPR109 on CD103^+^ DCs. Segmented filamentous bacteria (SFB) induce serum amyloid A (SAA) production from gut epithelial cells and stimulates CX3CR1^+^ DCs to promote Th17 cell development in the gut, the latter process could also be induced by commensal organism-derived ATP stimulation. *Clostridium* strains provide an environment rich in TGF-β that induces IL-10-producing colonic Treg Cells. *Bacteroides fragilis* (*B. fragilis*) promotes the differentiation and function Treg via its immunomodulatory molecule polysaccharide A (PSA), where Toll-like receptor 2 (TLR2) signaling is required. TLR2 ligand could also stimulate Tfh development in the germinal center to facilitate IgA production by B cells. Taken together, IL-17 and IL-22 produced by Th17, IL-10 and TGF-β produced by Treg cells, and IgA in the gut lumen constitute an immune barrier in maintaining gut homeostasis in steady state.

**Figure 3:**
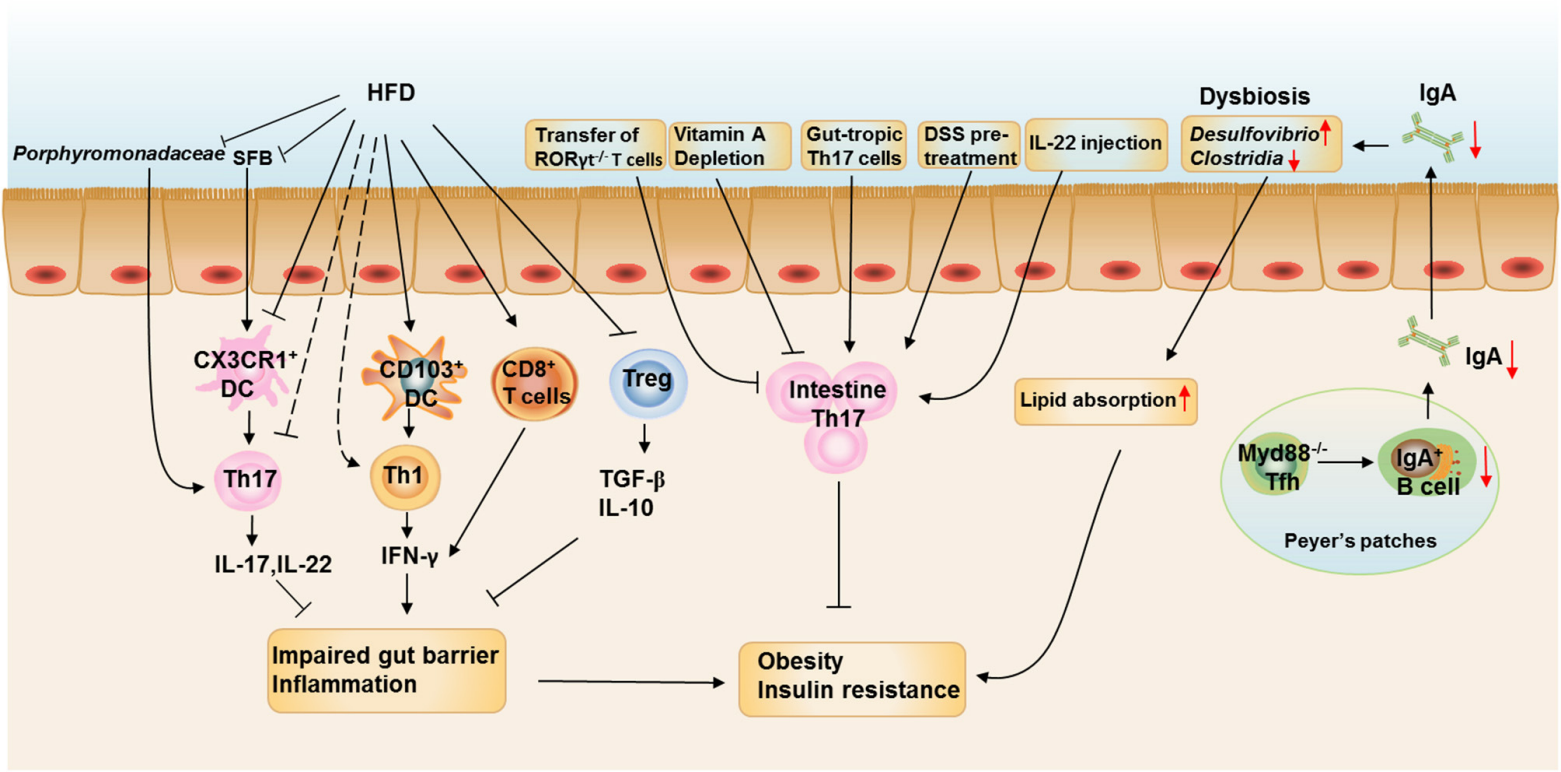
The roles of intestinal T cell immune responses in the development of obesity. HFD feeding reduces the proportions and numbers of RORγt^+^Th17 and Treg cells and increases the proportion of IFN-γ^+^ Th1 cells and CD8^+^ T cells in the SILP. HFD feeding also enhances the ability of CD103^+^ DCs to induce Th1 differentiation and inhibits the ability of CX3CR1^+^ cells to induce Th17 differentiation. The reduction of *Porphyromonadaceae* and *SFB* in the ileum at the onset of HFD feeding could be responsible for the reduction of intestinal Th17 cells. The increased IFN-γ production by Th1 and CD8^+^T cells impairs the gut barrier function and induces intestinal inflammation, which is aggravated by decreased production of IL-17, IL-22, TGF-β, and IL-10 from Th17 and Treg cells. Depletion of vitamin A in obese mice further reduced the proportion of Th17 cells in the small intestine, leading to increased body weight gain and insulin resistance and the metabolic disorders could be ameliorated by adoptive transfer of *in vitro*-differentiated gut-tropic Th17 cells to obese mice. Increasing Th17 cell proportion or function in the intestine by pretreating mice with dextran sodium sulfate or IL-22 administration also prevents HFD-induced glucose intolerance and insulinemia. However, Rag1^−/−^ mice adoptive transferred with T cells from RORγt^−/−^ mice leads to increased body weight gain and insulin resistance when compared with those adoptive transferred with wild-type T cells. IgA is also important in maintaining metabolic homeostasis by modulating gut microbiota homeostasis, while T cell-specific MyD88 knockout results in abnormal IgA antibody responses and an altered microbial gut community, leading to more severe HFD- and age-associated metabolic disorders.

In this review, we discuss the connection between intestinal local environments influenced by diets, specific microbes, metabolites, and gut T cell immune responses. We also highlight how the connection contributes to metabolic health and disease (Figure 2). We conclude that the imbalance of intestinal flora and modified APCs induced by HFD feeding directly or indirectly contribute to the dysregulation of T cell responses in the intestine, with increased Th1 and CD8^+^T cells as well as decreased Th17 and Treg cells. These T cell immune turbulence leads to intestinal inflammation and impaired gut barrier function, which contributes to subsequent obesity and insulin resistance. Besides, dysbiosis caused by defects in Tfh development and IgA production also critically involved in the HFD- and age-assciated obesity (Figure 3).

While accumulating evidence strongly suggests that intestinal T cells are potential therapeutic targets of metabolic diseases, many questions remain to be answered. It has been shown that, compared with younger people, the function of small intestinal CD4^+^ T cells in older people is reduced, concurrently with increased spontaneous cell death (95). However, it is currently unknown whether these changes contribute to aging-associated alterations in intestinal and metabolic homeostasis. Besides, cold exposure markedly shifts the composition of the gut microbiota, which contributes to thermogenesis and enhanced insulin sensitivity (135). Since gut microbiota is tightly associated with T cell immune responses (57) and thermogenesis is thought to boost mucosal immunity (136), it is worth determining whether intestinal T cells are involved in cold-induced thermogenesis. In addition, the ecology of gut microbiota and the composition of the intestine immune system vary according to the intestinal segments (48, 137). Therefore, the contributions of the interplay between microbiota and immune system in the gut to metabolic diseases may be intestinal segment-dependent and thus location distinct. What is more, controlling dysbiosis of gut microbiota by adaptive immunity is critically involved in the improvement of metabolic features, suggesting that vaccination may be considered as a possible therapeutic approach for T2D (36). Finally, a better understanding of the key cellular components of the intestinal T cell immune responses and their regulatory network, and how they control systemic metabolic homeostasis will allow researchers to design improved targeted therapies to treat intestinal and metabolic disorders.

## Author Contributions

HZ organized and wrote the draft. LW participated in the draft writing. FL revised the whole manuscript and offered constructive comments. All authors contributed to the article and approved the submitted version.

## Conflict of Interest

The authors declare that the research was conducted in the absence of any commercial or financial relationships that could be construed as a potential conflict of interest.

## References

[1] Tartof S Qian L Hong V Wei R Nadjafi R Fischer H. Obesity and mortality among patients diagnosed with COVID-19: results from an integrated Health Care Organization. Ann Int Med. (2020) 173:773–81. 10.7326/M20-3742 32783686 PMC7429998

[2] Popkin BM Du S Green WD Beck MA Algaith T Herbst CH. Individuals with obesity and COVID-19: a global perspective on the epidemiology and biological relationships. Obes Rev. (2020) 21:e13128. 10.1111/obr.13128 32845580 PMC7461480

[3] Zhou H Liu F. Regulation, communication, and functional roles of adipose tissue-resident CD4+ T cells in the control of metabolic homeostasis. Front Immunol. (2018) 9:1961. 10.3389/fimmu.2018.01961 30233575 PMC6134258

[4] Field CS Baixauli F Kyle RL Puleston DJ Cameron AM Sanin DE. Mitochondrial integrity regulated by lipid metabolism is a cell-intrinsic checkpoint for treg suppressive function. Cell Metab. (2020) 31:422–37.e5. 10.1016/j.cmet.2019.11.021 31883840 PMC7001036

[5] Garidou L Pomie C Klopp P Waget A Charpentier J Aloulou M. The gut microbiota regulates intestinal CD4 T cells expressing RORgammat and controls metabolic disease. Cell Metab. (2015) 22:100–12. 10.1016/j.cmet.2015.06.001 26154056

[6] Luck H Tsai S Chung J Clemente-Casares X Ghazarian M Revelo XS. Regulation of obesity-related insulin resistance with gut anti-inflammatory agents. Cell Metab. (2015) 21:527–42. 10.1016/j.cmet.2015.03.001 25863246

[7] Hong CP Park A Yang BG Yun CH Kwak MJ Lee GW. Gut-specific delivery of T-helper 17 cells reduces obesity and insulin resistance in mice. Gastroenterology. (2017) 152:1998–2010. 10.1053/j.gastro.2017.02.016 28246016

[8] Cerboni S Jeremiah N Gentili M Gehrmann U Conrad C Stolzenberg MC. Intrinsic antiproliferative activity of the innate sensor STING in T lymphocytes. J Exp Med. (2017) 214:1769–85. 10.1084/jem.20161674 28484079 PMC5461003

[9] Bollrath J Powrie FM. Controlling the frontier: regulatory T-cells and intestinal homeostasis. Semin Immunol. (2013) 25:352–7. 10.1016/j.smim.2013.09.002 24184013

[10] Littman DR Rudensky AY. Th17 and regulatory T cells in mediating and restraining inflammation. Cell. (2010) 140:845–58. 10.1016/j.cell.2010.02.021 20303875

[11] Shale M Schiering C Powrie F. CD4(+) T-cell subsets in intestinal inflammation. Immunol Rev. (2013) 252:164–82. 10.1111/imr.12039 23405904 PMC3736165

[12] Zhou H Wang Y Lian Q Yang B Ma Y Wu X. Differential IL-10 production by DCs determines the distinct adjuvant effects of LPS and PTX in EAE induction. Eur J Immunol. (2014) 44:1352–62. 10.1002/eji.201343744 24496948

[13] Lighvani AA Frucht DM Jankovic D Yamane H Aliberti J Hissong BD. T-bet is rapidly induced by interferon-gamma in lymphoid and myeloid cells. Proc Natl Acad Sci USA. (2001) 98:15137–42. 10.1073/pnas.261570598 11752460 PMC64996

[14] Johnston RJ Poholek AC DiToro D Yusuf I Eto D Barnett B. Bcl6 and Blimp-1 are reciprocal and antagonistic regulators of T follicular helper cell differentiation. Science. (2009) 325:1006–10. 10.1126/science.1175870 19608860 PMC2766560

[15] He L Gu W Wang M Chang X Sun X Zhang Y. Extracellular matrix protein 1 promotes follicular helper T cell differentiation and antibody production. Proc Natl Acad Sci USA. (2018) 115:8621–6. 10.1073/pnas.1801196115 30087185 PMC6112729

[16] Kubinak JL Petersen C Stephens WZ Soto R Bake E O'Connell RM. MyD88 signaling in T cells directs IgA-mediated control of the microbiota to promote health. Cell Host Microbe. (2015) 17:153–63. 10.1016/j.chom.2014.12.009 25620548 PMC4451207

[17] Toubal A Kiaf B Beaudoin L Cagninacci L Rhimi M Fruchet B. Mucosal-associated invariant T cells promote inflammation and intestinal dysbiosis leading to metabolic dysfunction during obesity. Nature Communications. (2020) 11:3755. 10.1038/s41467-020-17307-0 32709874 PMC7381641

[18] Lynch L Nowak M Varghese B Clark J Hogan A Toxavidis V. Adipose tissue invariant NKT cells protect against diet-induced obesity and metabolic disorder through regulatory cytokine production. Immunity. (2012) 37:574–87. 10.1016/j.immuni.2012.06.016 22981538 PMC4991771

[19] Blüher M. Obesity: global epidemiology and pathogenesis. Nat Rev Endocrinol. (2019) 15:288–98. 10.1038/s41574-019-0176-8 30814686

[20] Motwani M Pesiridis S Fitzgerald KA. DNA sensing by the cGAS-STING pathway in health and disease. Nat Rev Genet. (2019) 20:657–74. 10.1038/s41576-019-0151-1 31358977

[21] Yanovski J. Obesity: trends in underweight and obesity - scale of the problem. Nat Rev Endocrinol. (2018) 14:5–6. 10.1038/nrendo.2017.157 29170540 PMC5800307

[22] Pastorelli L De Salvo C Mercado J Vecchi M Pizarro T. Central role of the gut epithelial barrier in the pathogenesis of chronic intestinal inflammation: lessons learned from animal models and human genetics. Front Immunol. (2013) 4:280. 10.3389/fimmu.2013.00280 24062746 PMC3775315

[23] Wang X Ota N Manzanillo P Kates L Zavala-Solorio J Eidenschenk C. Interleukin-22 alleviates metabolic disorders and restores mucosal immunity in diabetes. Nature. (2014) 514:237–41. 10.1038/nature13564 25119041

[24] Forchielli ML Walker WA. The role of gut-associated lymphoid tissues and mucosal defence. Br J Nutr. (2005) 93(Suppl. 1):S41–8. 10.1079/BJN20041356 15877894

[25] Mowat AM. Anatomical basis of tolerance and immunity to intestinal antigens. Nat Rev Immunol. (2003) 3:331–41. 10.1038/nri1057 12669023

[26] Reboldi A Cyster J. Peyer's patches: organizing B-cell responses at the intestinal frontier. Immunol Rev. (2016) 271:230–45. 10.1111/imr.12400 27088918 PMC4835804

[27] Niess J Brand S Gu X Landsman L Jung S McCormick B. CX3CR1-mediated dendritic cell access to the intestinal lumen and bacterial clearance. Science. (2005) 307:254–8. 10.1126/science.1102901 15653504

[28] Farache J Koren I Milo I Gurevich I Kim KW Zigmond E. Luminal bacteria recruit CD103+ dendritic cells into the intestinal epithelium to sample bacterial antigens for presentation. Immunity. (2013) 38:581–95. 10.1016/j.immuni.2013.01.009 23395676 PMC4115273

[29] Hamada H Hiroi T Nishiyama Y Takahashi H Masunaga Y Hachimura S. Identification of multiple isolated lymphoid follicles on the antimesenteric wall of the mouse small intestine. J Immunol. (2002) 168:57–64. 10.4049/jimmunol.168.1.57 11751946

[30] Lorenz RG Chaplin DD McDonald KG McDonough JS Newberry RD. Isolated lymphoid follicle formation is inducible and dependent upon lymphotoxin-sufficient B lymphocytes, lymphotoxin beta receptor, and TNF receptor I function. J Immunol. (2003) 170:5475–82. 10.4049/jimmunol.170.11.5475 12759424

[31] Ussar S Griffin NW Bezy O Fujisaka S Vienberg S Softic S. Interactions between gut microbiota, host genetics and diet modulate the predisposition to obesity and metabolic syndrome. Cell Metab. (2015) 22:516–30. 10.1016/j.cmet.2015.07.007 26299453 PMC4570502

[32] Lalazar G Mizrahi M Turgeman I Adar T Ben Ya'acov A Shabat Y. Oral administration of OKT3 MAb to patients with NASH, promotes regulatory T-cell induction, and alleviates insulin resistance: results of a phase IIa blinded placebo-controlled trial. J Clin Immunol. (2015) 35:399–407. 10.1007/s10875-015-0160-6 25876706

[33] Lefrancois L. Phenotypic complexity of intraepithelial lymphocytes of the small intestine. J Immunol. (1991) 147:1746–51. 1716278

[34] Muller S Buhler-Jungo M Mueller C. Intestinal intraepithelial lymphocytes exert potent protective cytotoxic activity during an acute virus infection. J Immunol. (2000) 164:1986–94. 10.4049/jimmunol.164.4.1986 10657649

[35] Lefrancois L Puddington L. Intestinal and pulmonary mucosal T cells: local heroes fight to maintain the status quo. Annu Rev Immunol. (2006) 24:681–704. 10.1146/annurev.immunol.24.021605.090650 16551263

[36] Pomie C Blasco-Baque V Klopp P Nicolas S Waget A Loubieres P. Triggering the adaptive immune system with commensal gut bacteria protects against insulin resistance and dysglycemia. Mol Metab. (2016) 5:392–403. 10.1016/j.molmet.2016.03.004 27257599 PMC4877664

[37] Le Chatelier E Nielsen T Qin J Prifti E Hildebrand F Falony G. Richness of human gut microbiome correlates with metabolic markers. Nature. (2013) 500:541–6. 10.1038/nature12506 23985870

[38] Sabat R Ouyang W Wolk K. Therapeutic opportunities of the IL-22-IL-22R1 system. Nat Rev Drug Discov. (2014) 13:21–38. 10.1038/nrd4176 24378801

[39] Wu J Chen YJ Dobbs N Sakai T Liou J Miner JJ. STING-mediated disruption of calcium homeostasis chronically activates ER stress and primes T cell death. J Exp Med. (2019) 216:867–83. 10.1084/jem.20182192 30886058 PMC6446864

[40] Wu C Yosef N Thalhamer T Zhu C Xiao S Kishi Y. Induction of pathogenic TH17 cells by inducible salt-sensing kinase SGK1. Nature. (2013) 496:513–7. 10.1038/nature11984 23467085 PMC3637879

[41] Khovidhunkit SO Suwantuntula T Thaweboon S Mitrirattanakul S Chomkhakhai U Khovidhunkit W. Xerostomia, hyposalivation, and oral microbiota in type 2 diabetic patients: a preliminary study. J Med Assoc Thai. (2009) 92:1220–8. 19772183

[42] Rodrigues C Rodrigues M Henriques M. Candida sp. infections in patients with diabetes mellitus. J Clin Med. (2019) 8:76. 10.3390/jcm8010076 30634716 PMC6352194

[43] Hernandez A Kitz A Wu C Lowther D Rodriguez D Vudattu N. Sodium chloride inhibits the suppressive function of FOXP3+ regulatory T cells. J Clin Investig. (2015) 125:4212–22. 10.1172/JCI81151 26524592 PMC4639983

[44] Pandolfi JB Ferraro AA Sananez I Gancedo MC Baz P Billordo LA. ATP-induced inflammation drives tissue-resident Th17 cells in metabolically unhealthy obesity. J Immunol. (2016) 196:3287–96. 10.4049/jimmunol.1502506 26951799

[45] Chackelevicius CM Gambaro SE Tiribelli C Rosso N. Th17 involvement in nonalcoholic fatty liver disease progression to non-alcoholic steatohepatitis. World J Gastroenterol. (2016) 22:9096–103. 10.3748/wjg.v22.i41.9096 27895397 PMC5107591

[46] Endo Y Asou HK Matsugae N Hirahara K Shinoda K Tumes DJ. Obesity drives Th17 cell differentiation by inducing the lipid metabolic kinase, ACC1. Cell Rep. (2015) 12:1042–55. 10.1016/j.celrep.2015.07.014 26235623

[47] Winer S Paltser G Chan Y Tsui H Engleman E Winer D. Obesity predisposes to Th17 bias. Eur J Immunol. (2009) 39:2629–35. 10.1002/eji.200838893 19662632

[48] Mowat AM Agace WW. Regional specialization within the intestinal immune system. Nat Rev Immunol. (2014) 14:667–85. 10.1038/nri3738 25234148

[49] Cani PD Bibiloni R Knauf C Waget A Neyrinck AM Delzenne NM. Changes in gut microbiota control metabolic endotoxemia-induced inflammation in high-fat diet-induced obesity and diabetes in mice. Diabetes. (2008) 57:1470–81. 10.2337/db07-1403 18305141

[50] Miranda P De Palma G Serkis V Lu J Louis-Auguste M McCarville J. High salt diet exacerbates colitis in mice by decreasing Lactobacillus levels and butyrate production. Microbiome. (2018) 6:57. 10.1186/s40168-018-0433-4 29566748 PMC5865374

[51] Gulen MF Koch U Haag SM Schuler F Apetoh L Villunger A. Signalling strength determines proapoptotic functions of STING. Nat Commun. (2017) 8:427. 10.1038/s41467-017-00573-w 28874664 PMC5585373

[52] Ridaura VK Faith JJ Rey FE Cheng J Duncan AE Kau AL. Gut microbiota from twins discordant for obesity modulate metabolism in mice. Science. (2013) 341:1241214. 10.1126/science.1241214 24009397 PMC3829625

[53] Petersen C Bell R Klag KA Lee SH Soto R Ghazaryan A. T cell-mediated regulation of the microbiota protects against obesity. Science. (2019) 365:eaat9351. 10.1126/science.aat9351 31346040 PMC7294966

[54] Araujo JR Tazi A Burlen-Defranoux O Vichier-Guerre S Nigro G Licandro H. Fermentation products of commensal bacteria alter enterocyte lipid metabolism. Cell Host Microbe. (2020) 27:358–75 e7. 10.1016/j.chom.2020.01.028 32101704

[55] Duparc T Plovier H Marrachelli VG Van Hul M Essaghir A Stahlman M. Hepatocyte MyD88 affects bile acids, gut microbiota and metabolome contributing to regulate glucose and lipid metabolism. Gut. (2017) 66:620–32. 10.1136/gutjnl-2015-310904 27196572 PMC5529962

[56] Alexander KL Targan SR Elson CO III. Microbiota activation and regulation of innate and adaptive immunity. Immunol Rev. (2014) 260:206–20. 10.1111/imr.12180 24942691 PMC4080089

[57] Belkaid Y Hand TW. Role of the microbiota in immunity and inflammation. Cell. (2014) 157:121–41. 10.1016/j.cell.2014.03.011 24679531 PMC4056765

[58] Ivanov II Frutos Rde L Manel N Yoshinaga K Rifkin DB. Specific microbiota direct the differentiation of IL-17-producing T-helper cells in the mucosa of the small intestine. Cell Host Microbe. (2008) 4:337–49. 10.1016/j.chom.2008.09.009 18854238 PMC2597589

[59] Ivanov I Atarashi K Manel N Brodie E Shima T Karaoz U. Induction of intestinal Th17 cells by segmented filamentous bacteria. Cell. (2009) 139:485–98. 10.1016/j.cell.2009.09.033 19836068 PMC2796826

[60] Wilck N Matus M Kearney S Olesen S Forslund K Bartolomaeus H. Salt-responsive gut commensal modulates T17 axis and disease. Nature. (2017) 551:585–9. 10.1038/nature24628 29143823 PMC6070150

[61] Siracusa F Schaltenberg N Villablanca E Huber S Gagliani N. Dietary habits and intestinal immunity: from food intake to CD4 T cells. Front Immunol. (2018) 9:3177. 10.3389/fimmu.2018.03177 30697217 PMC6340974

[62] Plovier H Everard A Druart C Depommier C Van Hul M Geurts L. A purified membrane protein from Akkermansia muciniphila or the pasteurized bacterium improves metabolism in obese and diabetic mice. Nat Med. (2017) 23:107–13. 10.1038/nm.4236 27892954

[63] Everard A Belzer C Geurts L Ouwerkerk JP Druart C Bindels LB. Cross-talk between Akkermansia muciniphila and intestinal epithelium controls diet-induced obesity. Proc Natl Acad Sci USA. (2013) 110:9066–71. 10.1073/pnas.1219451110 23671105 PMC3670398

[64] Feuerer M Hill JA Kretschmer K von Boehmer H Mathis D Benoist C. Genomic definition of multiple ex vivo regulatory T cell subphenotypes. Proc Natl Acad Sci USA. (2010) 107:5919–24. 10.1073/pnas.1002006107 20231436 PMC2851866

[65] Nutsch K Chai J Ai T Russler-Germain E Feehley T Nagler C. Rapid and efficient generation of regulatory T cells to commensal antigens in the periphery. Cell reports. (2016) 17:206–20. 10.1016/j.celrep.2016.08.092 27681432 PMC5051580

[66] Atarashi K Tanoue T Shima T Imaoka A Kuwahara T Momose Y. Induction of colonic regulatory T cells by indigenous Clostridium species. Science. (2011) 331:337–41. 10.1126/science.1198469 21205640 PMC3969237

[67] Kim K Hong S Han D Yi J Jung J Yang B. Dietary antigens limit mucosal immunity by inducing regulatory T cells in the small intestine. Science. (2016) 351:858–63. 10.1126/science.aac5560 26822607

[68] Lathrop SK Bloom SM Rao SM Nutsch K Lio CW Santacruz N. Peripheral education of the immune system by colonic commensal microbiota. Nature. (2011) 478:250–4. 10.1038/nature10434 21937990 PMC3192908

[69] Round JL Mazmanian SK. Inducible Foxp3+ regulatory T-cell development by a commensal bacterium of the intestinal microbiota. Proc Natl Acad Sci USA. (2010) 107:12204–9. 10.1073/pnas.0909122107 20566854 PMC2901479

[70] Crotty S. Follicular helper CD4 T cells (TFH). Annu Rev Immunol. (2011) 29:621–63. 10.1146/annurev-immunol-031210-101400 21314428

[71] Hofmann AF Hagey LR. Key discoveries in bile acid chemistry and biology and their clinical applications: history of the last eight decades. J Lipid Res. (2014) 55:1553–95. 10.1194/jlr.R049437 24838141 PMC4109754

[72] Duboc H Rajca S Rainteau D Benarous D Maubert MA Quervain E. Connecting dysbiosis, bile-acid dysmetabolism and gut inflammation in inflammatory bowel diseases. Gut. (2013) 62:531–9. 10.1136/gutjnl-2012-302578 22993202

[73] Ma H Patti ME. Bile acids, obesity, and the metabolic syndrome. Best Pract Res Clin Gastroenterol. (2014) 28:573–83. 10.1016/j.bpg.2014.07.004 25194176 PMC4159616

[74] Yao L Seaton S Ndousse-Fetter S Adhikari A DiBenedetto N Mina A. A selective gut bacterial bile salt hydrolase alters host metabolism. eLife. (2018) 7:e37182. 10.7554/eLife.37182 30014852 PMC6078496

[75] Pols TWH Puchner T Korkmaz HI Vos M Soeters MR de Vries CJM. Lithocholic acid controls adaptive immune responses by inhibition of Th1 activation through the Vitamin D receptor. PLoS ONE. (2017) 12:e0176715. 10.1371/journal.pone.0176715 28493883 PMC5426628

[76] Hang S Paik D Yao L Kim E Trinath J Lu J. Bile acid metabolites control TH17 and Treg cell differentiation. Nature. (2019) 576:143–8. 10.1038/s41586-019-1785-z 31776512 PMC6949019

[77] Jaensson-Gyllenback E Kotarsky K Zapata F Persson EK Gundersen TE Blomhoff R. Bile retinoids imprint intestinal CD103+ dendritic cells with the ability to generate gut-tropic T cells. Mucosal Immunol. (2011) 4:438–47. 10.1038/mi.2010.91 21289617 PMC3130189

[78] Kang SG Wang C Matsumoto S Kim CH. High and low vitamin A therapies induce distinct FoxP3+ T-cell subsets and effectively control intestinal inflammation. Gastroenterology. (2009) 137:1391–402 e1–6. 10.1053/j.gastro.2009.06.063 19632226 PMC2757541

[79] Agace WW Persson EK. How vitamin A metabolizing dendritic cells are generated in the gut mucosa. Trends Immunol. (2012) 33:42–8. 10.1016/j.it.2011.10.001 22079120

[80] Fernandes J Su W Rahat-Rozenbloom S Wolever TM Comelli EM. Adiposity, gut microbiota and faecal short chain fatty acids are linked in adult humans. Nutr Diabetes. (2014) 4:e121. 10.1038/nutd.2014.23 24979150 PMC4079931

[81] Thorburn AN Macia L Mackay CR. Diet, metabolites, and “western-lifestyle” inflammatory diseases. Immunity. (2014) 40:833–42. 10.1016/j.immuni.2014.05.014 24950203

[82] Smith P Howitt M Panikov N Michaud M Gallini C Bohlooly-Y M. The microbial metabolites, short-chain fatty acids, regulate colonic Treg cell homeostasis. Science. (2013) 341:569–73. 10.1126/science.1241165 23828891 PMC3807819

[83] Singh N Gurav A Sivaprakasam S Brady E Padia R Shi H. Activation of Gpr109a, receptor for niacin and the commensal metabolite butyrate, suppresses colonic inflammation and carcinogenesis. Immunity. (2014) 40:128–39. 10.1016/j.immuni.2013.12.007 24412617 PMC4305274

[84] Gao Z Yin J Zhang J Ward RE Martin RJ Lefevre M. Butyrate improves insulin sensitivity and increases energy expenditure in mice. Diabetes. (2009) 58:1509–17. 10.2337/db08-1637 19366864 PMC2699871

[85] Arpaia N Campbell C Fan X Dikiy S van der Veeken J deRoos P. Metabolites produced by commensal bacteria promote peripheral regulatory T-cell generation. Nature. (2013) 504:451–5. 10.1038/nature12726 24226773 PMC3869884

[86] Kespohl M Vachharajani N Luu M Harb H Pautz S Wolff S. The microbial metabolite butyrate induces expression of Th1-associated factors in CD4 T cells. Front Immunol. (2017) 8:1036. 10.3389/fimmu.2017.01036 28894447 PMC5581317

[87] Li Y Innocentin S Withers D Roberts N Gallagher A Grigorieva E. Exogenous stimuli maintain intraepithelial lymphocytes via aryl hydrocarbon receptor activation. Cell. (2011) 147:629–40. 10.1016/j.cell.2011.09.025 21999944

[88] Quintana FJ Basso AS Iglesias AH Korn T Farez MF Bettelli E. Control of T(reg) and T(H)17 cell differentiation by the aryl hydrocarbon receptor. Nature. (2008) 453:65–71. 10.1038/nature06880 18362915

[89] Kiss EA Vonarbourg C Kopfmann S Hobeika E Finke D Esser C. Natural aryl hydrocarbon receptor ligands control organogenesis of intestinal lymphoid follicles. Science. (2011) 334:1561–5. 10.1126/science.1214914 22033518

[90] Lin C Theodorides M McDaniel A Tordoff M Zhang Q Li X. QTL analysis of dietary obesity in C57BL/6byj X 129P3/J F2 mice: diet- and sex-dependent effects. PLoS ONE. (2013) 8:e68776. 10.1371/journal.pone.0068776 23922663 PMC3726688

[91] Moyer B Rojas I Kerley-Hamilton J Nemani K Trask H Ringelberg C. Obesity and fatty liver are prevented by inhibition of the aryl hydrocarbon receptor in both female and male mice. Nutr Res. (2017) 44:38–50. 10.1016/j.nutres.2017.06.002 28821316 PMC5569910

[92] Korecka A Dona A Lahiri S Tett A Al-Asmakh M Braniste V. Bidirectional communication between the Aryl hydrocarbon Receptor (AhR) and the microbiome tunes host metabolism. NPJ Biofilms Microbiomes. (2016) 2:16014. 10.1038/npjbiofilms.2016.14 28721249 PMC5515264

[93] Suzuki H. Age-dependent changes in intraepithelial lymphocytes. (IELs) of the small intestine, cecum, and colon from young adult to aged mice. Arch Gerontol Geriatr. (2012) 55:261–70. 10.1016/j.archger.2011.07.009 21840070

[94] Morimoto M Saito C Muto C Akamatsu Y Chiba T Abe T. Impairment of host resistance to helminthes with age in murine small intestine. Parasite Immunol. (2015) 37:171–9. 10.1111/pim.12170 25545318

[95] Dillon SM Liu J Purba CM Christians AJ Kibbie JJ Castleman MJ. Age-related alterations in human gut CD4 T cell phenotype, T helper cell frequencies, and functional responses to enteric bacteria. J Leukoc Biol. (2020) 107:119–32. 10.1002/JLB.5A0919-177RR 31573727

[96] DeBoer M Lima A Oría R Scharf R Moore S Luna M. Early childhood growth failure and the developmental origins of adult disease: do enteric infections and malnutrition increase risk for the metabolic syndrome? Nutr Rev. (2012) 70:642–53. 10.1111/j.1753-4887.2012.00543.x 23110643 PMC3493112

[97] Semba RD Trehan I Li X Salem N Jr. Moaddel R. Low serum omega-3 and omega-6 polyunsaturated fatty acids and other metabolites are associated with poor linear growth in young children from rural Malawi. Am J Clin Nutr. (2017) 106:1490–9. 10.3945/ajcn.117.164384 29070563 PMC5698844

[98] Gehrig J Venkatesh S Chang H Hibberd M Kung V Cheng J. Effects of microbiota-directed foods in gnotobiotic animals and undernourished children. Science. (2019) 365:eaau4732. 10.1126/science.aau4732 31296738 PMC6683325

[99] Subramanian S Huq S Yatsunenko T Haque R Mahfuz M Alam M. Persistent gut microbiota immaturity in malnourished Bangladeshi children. Nature. (2014) 510:417–21. 10.1038/nature13421 24896187 PMC4189846

[100] Bourke C Berkley J Prendergast A. Immune dysfunction as a cause and consequence of malnutrition. Trends Immunol. (2016) 37:386–98. 10.1016/j.it.2016.04.003 27237815 PMC4889773

[101] Collins N Han SJ Enamorado M Link VM Huang B Moseman EA. The bone marrow protects and optimizes immunological memory during dietary restriction. Cell. (2019) 178:1088–101 e15. 10.1016/j.cell.2019.07.049 31442402 PMC6818271

[102] Redman LM Smith SR Burton JH Martin CK Il'yasova D Ravussin E. Metabolic slowing and reduced oxidative damage with sustained caloric restriction support the rate of living and oxidative damage theories of aging. Cell Metab. (2018) 27:805–15 e4. 10.1016/j.cmet.2018.02.019 29576535 PMC5886711

[103] Cignarella F Cantoni C Ghezzi L Salter A Dorsett Y Chen L. Intermittent fasting confers protection in CNS autoimmunity by altering the gut microbiota. Cell Metab. (2018) 27:1222–35 e6. 10.1016/j.cmet.2018.05.006 29874567 PMC6460288

[104] Nagai M Noguchi R Takahashi D Morikawa T Koshida K Komiyama S. Fasting-refeeding impacts immune cell dynamics and mucosal immune responses. Cell. (2019) 178:1072–87 e14. 10.1016/j.cell.2019.07.047 31442401

[105] Hu D Mao Y Xu G Liao W Ren J Yang H. Time-restricted feeding causes irreversible metabolic disorders and gut microbiota shift in pediatric mice. Pediatr Res. (2019) 85:518–26. 10.1038/s41390-018-0156-z 30188503 PMC6760561

[106] Hapfelmeier S Müller A Stecher B Kaiser P Barthel M Endt K. Microbe sampling by mucosal dendritic cells is a discrete, MyD88-independent step in DeltainvG S. Typhimurium colitis. J Exp Med. (2008) 205:437–50. 10.1084/jem.20070633 18268033 PMC2271026

[107] Varol C Vallon-Eberhard A Elinav E Aychek T Shapira Y Luche H. Intestinal lamina propria dendritic cell subsets have different origin and functions. Immunity. (2009) 31:502–12. 10.1016/j.immuni.2009.06.025 19733097

[108] Coombes JL Siddiqui KR Arancibia-Carcamo CV Hall J Sun CM Belkaid Y. A functionally specialized population of mucosal CD103+ DCs induces Foxp3+ regulatory T cells via a TGF-beta and retinoic acid-dependent mechanism. J Exp Med. (2007) 204:1757–64. 10.1084/jem.20070590 17620361 PMC2118683

[109] Tanoue T Honda K. Induction of Treg cells in the mouse colonic mucosa: a central mechanism to maintain host-microbiota homeostasis. Semin Immunol. (2012) 24:50–7. 10.1016/j.smim.2011.11.009 22172550

[110] Travis M Reizis B Melton A Masteller E Tang Q Proctor J. Loss of integrin alpha(v)beta8 on dendritic cells causes autoimmunity and colitis in mice. Nature. (2007) 449:361–5. 10.1038/nature06110 17694047 PMC2670239

[111] Uematsu S Fujimoto K Jang M Yang B Jung Y Nishiyama M. Regulation of humoral and cellular gut immunity by lamina propria dendritic cells expressing Toll-like receptor 5. Nature immunology. (2008) 9:769–76. 10.1038/ni.1622 18516037

[112] Laffont S Siddiqui KR Powrie F. Intestinal inflammation abrogates the tolerogenic properties of MLN CD103+ dendritic cells. Eur J Immunol. (2010) 40:1877–83. 10.1002/eji.200939957 20432234 PMC6108414

[113] Atarashi K Nishimura J Shima T Umesaki Y Yamamoto M Onoue M. ATP drives lamina propria TH17 cell differentiation. Nature. (2008) 455:808–12. 10.1038/nature07240 18716618

[114] Varol C Zigmond E Jung S. Securing the immune tightrope: mononuclear phagocytes in the intestinal lamina propria. Nat Rev Immunol. (2010) 10:415–26. 10.1038/nri2778 20498668

[115] Cani PD Amar J Iglesias MA Poggi M Knauf C Bastelica D. Metabolic endotoxemia initiates obesity and insulin resistance. Diabetes. (2007) 56:1761–72. 10.2337/db06-1491 17456850

[116] Chen H Zhang S Schwarzschild M Hernán M Willett W Ascherio A. Obesity and the risk of Parkinson's disease. Am J Epidemiol. (2004) 159:547–55. 10.1093/aje/kwh059 15003958

[117] Campos-Acuna J Elgueta D Pacheco R. T-cell-driven inflammation as a mediator of the gut-brain axis involved in Parkinson's Disease. Front Immunol. (2019) 10:239. 10.3389/fimmu.2019.00239 30828335 PMC6384270

[118] Li W Lu L Lu J Wang X Yang C Jin J. cGAS-STING-mediated DNA sensing maintains CD8 T cell stemness and promotes antitumor T cell therapy. Sci Transl Med. (2020) 12:eaay9013. 10.1126/scitranslmed.aay9013 32581136

[119] Ji Z Wu S Xu Y Qi J Su X Shen L. Obesity promotes EAE through IL-6 and CCL-2-mediated T cells infiltration. Front Immunol. (2019) 10:1881. 10.3389/fimmu.2019.01881 31507583 PMC6718738

[120] Duc D Vigne S Bernier-Latmani J Yersin Y Ruiz F Gaïa N. Disrupting myelin-specific Th17 cell gut homing confers protection in an adoptive transfer experimental autoimmune encephalomyelitis. Cell Rep. (2019) 29:378–90.e4. 10.1016/j.celrep.2019.09.002 31597098

[121] Su L Wu Z Chi Y Song Y Xu J Tan J. Mesenteric lymph node CD4(+) T lymphocytes migrate to liver and contribute to non-alcoholic fatty liver disease. Cell Immunol. (2019) 337:33–41. 10.1016/j.cellimm.2019.01.005 30770094

[122] Adams DH Eksteen B. Aberrant homing of mucosal T cells and extra-intestinal manifestations of inflammatory bowel disease. Nat Rev Immunol. (2006) 6:244–51. 10.1038/nri1784 16498453

[123] Wu J Dobbs N Yang K Yan N. Interferon-independent activities of mammalian STING mediate antiviral response and tumor immune evasion. Immunity. (2020) 53:115–26.e5. 10.1016/j.immuni.2020.06.009 32640258 PMC7365768

[124] de Krijger M Wildenberg ME de Jonge WJ Ponsioen CY. Return to sender: lymphocyte trafficking mechanisms as contributors to primary sclerosing cholangitis. J Hepatol. (2019) 71:603–15. 10.1016/j.jhep.2019.05.006 31108158

[125] Rousseaux C Lefebvre B Dubuquoy L Lefebvre P Romano O Auwerx J. Intestinal antiinflammatory effect of 5-aminosalicylic acid is dependent on peroxisome proliferator-activated receptor-gamma. J Exp Med. (2005) 201:1205–15. 10.1084/jem.20041948 15824083 PMC2213148

[126] Bereswill S Escher U Grunau A Kuhl AA Dunay IR Tamas A. Pituitary adenylate cyclase-activating polypeptide-A neuropeptide as novel treatment option for subacute ileitis in mice harboring a human gut microbiota. Front Immunol. (2019) 10:554. 10.3389/fimmu.2019.00554 30967875 PMC6438926

[127] Li J Sung CY Lee N Ni Y Pihlajamaki J Panagiotou G. Probiotics modulated gut microbiota suppresses hepatocellular carcinoma growth in mice. Proc Natl Acad Sci USA. (2016) 113:E1306–15. 10.1073/pnas.1518189113 26884164 PMC4780612

[128] Leber A Hontecillas R Zoccoli-Rodriguez V Ehrich M Chauhan J Bassaganya-Riera J. Exploratory studies with NX-13: oral toxicity and pharmacokinetics in rodents of an orally active, gut-restricted first-in-class therapeutic for IBD that targets NLRX1. Drug Chem Toxicol. (2019) 1−6. 10.1080/01480545.2019.1679828 31650868 PMC7182494

[129] Leber A Hontecillas R Zoccoli-Rodriguez V Bienert C Chauhan J Bassaganya-Riera J. Activation of NLRX1 by NX-13 alleviates inflammatory bowel disease through immunometabolic mechanisms in CD4(+) T cells. J Immunol. (2019) 203:3407–15. 10.4049/jimmunol.1900364 31694910 PMC6904519

[130] Leber A Hontecillas R Zoccoli-Rodriguez V Bassaganya-Riera J. Activation of LANCL2 by BT-11 ameliorates IBD by supporting regulatory T cell stability through immunometabolic mechanisms. Inflamm Bowel Dis. (2018) 24:1978–91. 10.1093/ibd/izy167 29718324 PMC6241665

[131] Leber A Hontecillas R Zoccoli-Rodriguez V Chauhan J Bassaganya-Riera J. Oral treatment with BT-11 ameliorates inflammatory bowel disease by enhancing regulatory T cell responses in the gut. J Immunol. (2019) 202:2095–104. 10.4049/jimmunol.1801446 30760618

[132] Ochi H Abraham M Ishikawa H Frenkel D Yang K Basso AS. Oral CD3-specific antibody suppresses autoimmune encephalomyelitis by inducing CD4+ CD25- LAP+ T cells. Nat Med. (2006) 12:627–35. 10.1038/nm1408 16715091

[133] You S Leforban B Garcia C Bach JF Bluestone JA Chatenoud L. Adaptive TGF-beta-dependent regulatory T cells control autoimmune diabetes and are a privileged target of anti-CD3 antibody treatment. Proc Natl Acad Sci USA. (2007) 104:6335–40. 10.1073/pnas.0701171104 17389382 PMC1851030

[134] Ilan Y Maron R Tukpah AM Maioli TU Murugaiyan G Yang K. Induction of regulatory T cells decreases adipose inflammation and alleviates insulin resistance in ob/ob mice. Proc Natl Acad Sci USA. (2010) 107:9765–70. 10.1073/pnas.0908771107 20445103 PMC2906892

[135] Chevalier C Stojanović O Colin Didier J Suarez-Zamorano N Tarallo V Veyrat-Durebex C. Gut microbiota orchestrates energy homeostasis during cold. Cell. (2015) 163:1360–74. 10.1016/j.cell.2015.11.004 26638070

[136] Liao WH Henneberg M Langhans W. Immunity-based evolutionary interpretation of diet-induced thermogenesis. Cell Metab. (2016) 23:971–9. 10.1016/j.cmet.2016.05.002 27304499

[137] Albenberg L Esipova TV Judge CP Bittinger K Chen J Laughlin A. Correlation between intraluminal oxygen gradient and radial partitioning of intestinal microbiota. Gastroenterology. (2014) 147:1055–63 e8. 10.1053/j.gastro.2014.07.020 25046162 PMC4252572

