# Corrigendum: Immunological Impact of Intestinal T Cells on Metabolic Diseases

**DOI:** 10.3389/fimmu.2021.682376

**Published:** 2021-04-14

**Authors:** Haiyan Zhou, Liwen Wang, Feng Liu

**Affiliations:** ^1^ National Clinical Research Center for Metabolic Diseases, Metabolic Syndrome Research Center, and Department of Metabolism and Endocrinology, The Second Xiangya Hospital of Central South University, Changsha, China; ^2^ Department of Pharmacology, University of Texas Health Science Center at San Antonio, San Antonio, TX, United States

**Keywords:** obesity, intestine, T cells, microbiota, dietary signals

In the published article, there was an error in affiliation 1. Instead of “Department of Metabolism and Endocrinology, National Clinical Research Center for Metabolic Diseases, Metabolic Syndrome Research Center, The Second Xiangya Hospital of Central South University, Changsha, China”, it should be “National Clinical Research Center for Metabolic Diseases, Metabolic Syndrome Research Center, and Department of Metabolism and Endocrinology, The Second Xiangya Hospital of Central South University, Changsha, China”.

The authors apologize for this error and state that this does not change the scientific conclusions of the article in any way. The original article has been updated.

